# Complex Reorientation Dynamics of Sizable Glass-Formers with Polar Rotors Revealed by Dielectric Spectroscopy

**DOI:** 10.1021/acs.jpclett.1c03088

**Published:** 2021-11-15

**Authors:** Marzena Rams-Baron, Beibei Yao, Shinian Cheng, Mateusz Dulski, Marian Paluch

**Affiliations:** † August Chełkowski Institute of Physics, University of Silesia, 75 Pulku Piechoty 1, 41-500 Chorzow, Poland; ‡ Silesian Center for Education and Interdisciplinary Research, 75 Pulku Piechoty 1a, 41-500 Chorzow, Poland; § Institute of Materials Engineering, University of Silesia, 75 Pulku Piechoty 1a, 41-500 Chorzow, Poland; ⊥ Silesian Center for Education and Interdisciplinary Research, 75 Pulku Piechoty 1a, 41-500 Chorzow, Poland

## Abstract

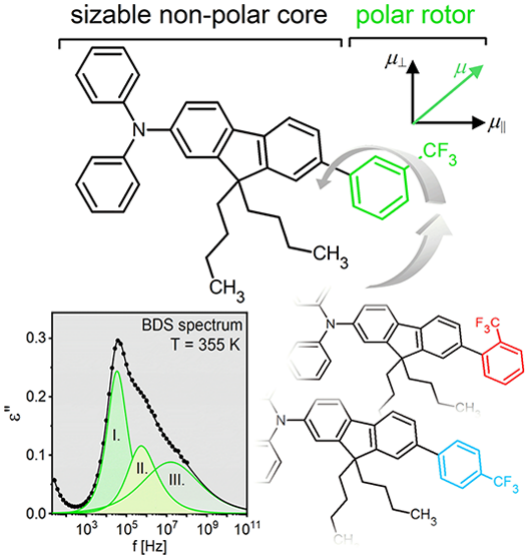

We present the results of dielectric measurements for three sizable glass-formers with identical nonpolar cores linked to various dipole-labeled rotors that shed new light on the picture of reorientation of anisotropic systems with significant moment of inertia revealed by broadband dielectric spectroscopy. The dynamics of sizable glass-formers formed by partially rigid molecular cores linked to small polar rotors in many respects differs from that of typical glass-formers. For instance, the extraordinarily large prefactors (τ_0_ > 10^–12^ s) in the Vogel–Fulcher–Tammann equation were found. The rich and highly diverse relaxation pattern was governed by the location of a dipole, its ability to rotate freely, and the degree of coupling to the motion of the entire sizable system.

Molecular dynamics of sizable molecules composed of many atoms arranged in a structure with rigid and flexible subunits represent a fundamental problem with significant technological implications. Broadband dielectric spectroscopy (BDS) can be used to gain insight into the rich dynamics of large molecules. The dielectrically probed fluctuations of molecular dipoles allow the study of molecular motions on many scales, from the internal rotations of side groups to whole molecule reorientations and even supramolecular networks rearrangements.^1−4^ The only necessary condition is the presence of the dipolar group in the material.

Gaining a better understanding of the motion of large systems is undoubtedly one of the most engaging scientific challenges of our time. Macromolecules, biological motors, and artificial machines are the research objects that stimulate the imagination and drive conceptual, experimental, and theoretical efforts in the field.^5,6^ However, basic knowledge about the structure–dynamics relations in the constituent molecules with desired functionality needs to be uncovered as a starting point. Therefore, we have proposed a new concept of sizable glass-forming materials^7,8^ with structural features corresponding to those found in applicable tempting materials (e.g., in optoelectronics) but at the same time allowing reference to the fundamental issues related to the reorientation dynamics of large, anisotropic, partially planar, and rigid molecular systems.

Sizable molecules cannot be simply regarded as entities containing many atoms (molar masses of *approx*. 600 g/mol, number of atoms > 80) that match a gap between the low-molecular-weight glass-formers and polymers, both well recognized by the dielectric community in the past. The size criterion, while appropriate, is not the most important. The circumstance that justifies the categorization as a separate class of glass-forming materials is unusual dielectric behavior, distinct from those observed for other groups of glass-formers. For instance, as will be grounded by the results discussed in this Letter, contrary to the most glass-forming liquids, the pre-exponential factor, τ_0_, in the Vogel–Fulcher–Tammann (VFT) equation parametrizing the correlation times for a sizable molecule’s reorientations and equated with the inverse attempt frequency for barrier crossing, substantially exceeds the typical phonon-like time-scale of 10^–14^ s. Recalling the Bauer’s expression,^9^ τ_0_ ∼ (2π*I*/*k*_B_*T*)^0.5^, which takes the moment of inertia, *I*, into account (*k*_B_ is a Boltzmann constant, *T* is temperature), the observed large values of τ_0_ imply that the inertia of these partially rigid molecules of considerable sizes determines to some extent their relaxation properties.

The consideration of large systems with significant moments of inertia leads to some questions regarding their rotational dynamics, most of which have not been directly addressed before. Thus, the results presented in this Letter are a wealth of new observations that may revise the scientific understanding of the dielectric response of large and partially rigid glass-formers. We present here dielectric results for three sizable molecules with an identical molecular core containing fluorene and diphenylamine motifs linked to a small polar unit. The partially planar framework of a sizable molecule is characterized by a certain degree of stiffness conditioned by the presence of many aromatic ring structures functionalized by flexible alkyl chains. From the perspective of dielectric research, it is essential that only one localized dipole contributes to the dielectric response. Considering this, the sizable molecule can be divided into two parts. The large nonpolar framework (assigned as M) is connected via a single C–C bond to the second segment formed by the phenyl ring with the trifluoromethylgroup attached (described as Ph-CF_3_). The small polar group −CF_3_ carries the dipole moment, which makes the motions of the whole system detectable by the BDS method. It is variably attached to the phenyl ring (in *ortho*, *meta*, or *para* positions). The corresponding sizable molecules are described as M-Ph-*ortho*-CF_3_, M-Ph-*meta*-CF_3_, and M-Ph-*para*-CF_3_, respectively. They were synthesized on request by TriMen Chemicals (Lodz, Poland) with a purity ≥ 97%. Their chemical structures are shown by the upper panels in Figure 1. Although the value of the dipole moment of the investigated isomers is similar, its orientation differs significantly in individual systems indicated by arrows on the upper panels in Figure 1a–c.

**Figure 1:**
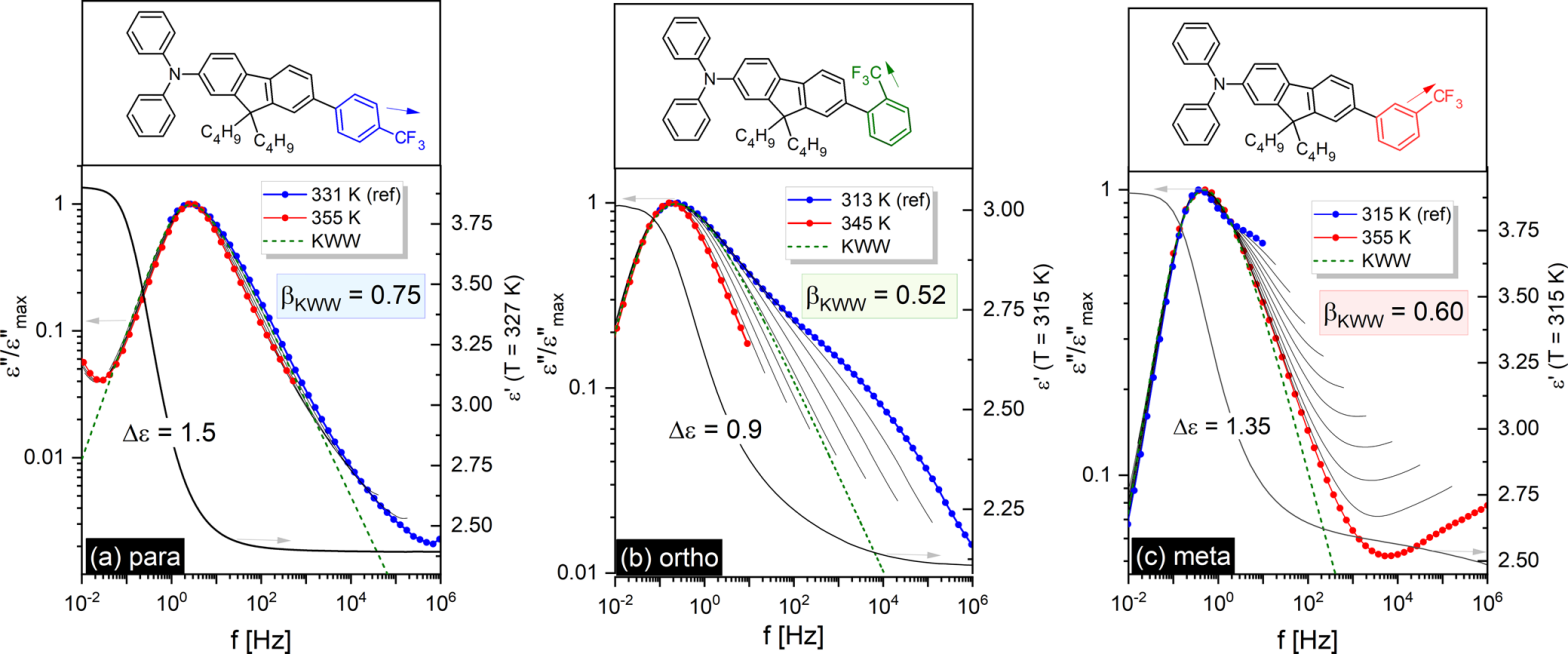
(Left axis) Masterplots constructed by horizontal shifting of normalized spectra on the one registered at *T* = 331 K for M-Ph-*para*-CF_3_ (a), 313 K for M-Ph-*ortho*-CF_3_ (b), and 315 K for M-Ph-*meta*-C*F*_3_(*c*). (Right axis) Representative ε′(*f*) spectrum and the corresponding dielectric strength Δε. Upper panels show the chemical structures of sizable glass-formers (the arrow approximates the direction of the dipole moment).

The investigated sizable molecules can be simply illustrated as a rigid unit with a smaller, rapidly rotating element (rotor) bound by a single covalent bond to the rigid part. Such an idealized model perfectly illustrates that, from the dynamics perspective, the sizable systems offer the unique combination of molecular motions on various scales involving whole molecule reorientations (i) and internal rotations (ii). To follow their reorientation dynamics, we measured the complex dielectric permittivity ε*(*f*) = *ε*′(*f*) – *iε*″(*f*) of melt-quenched samples using the Novocontrol Alpha analyzer (see Supporting Information for experimental details). The characteristic dynamic parameters are summarized in Table S1. The representative dielectric loss spectra presented in Figure 2 show that the relaxation behaviors of particular isomers vary significantly.

**Figure 2:**
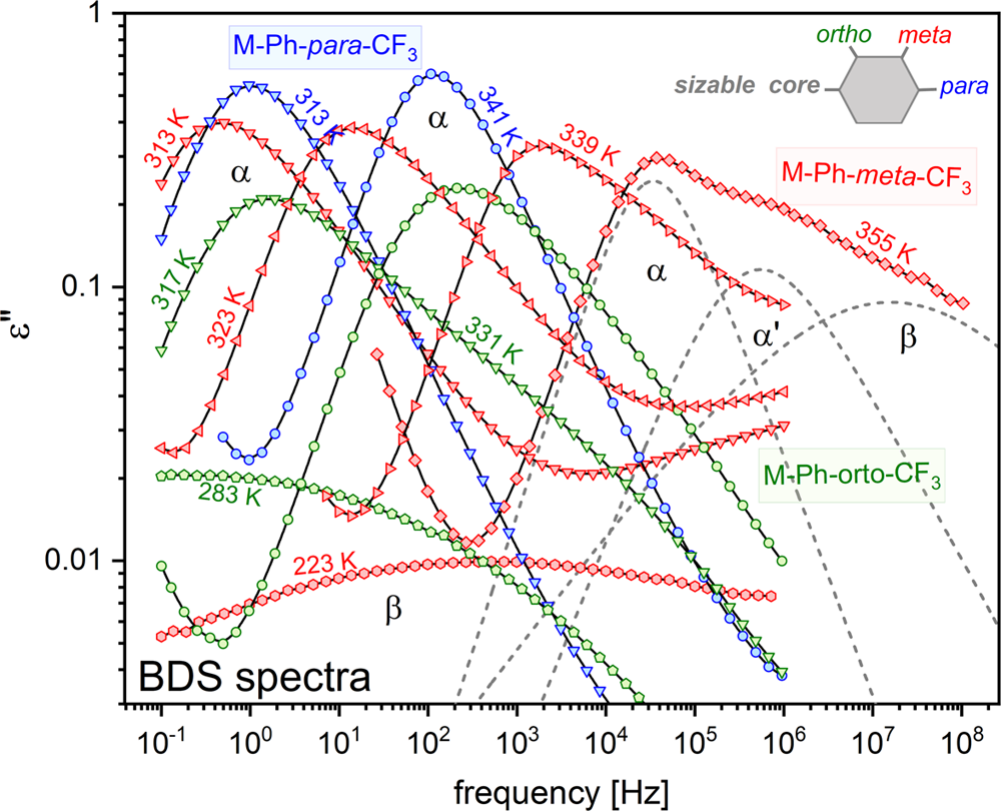
Representative dielectric loss spectra of sizable glass-formers with the same large structures differing only in the position of −CF_3_ group (blue symbols, *para* isomer; green, *ortho*; red, *meta*). For the spectrum recorded at 355 K, the components are disentangled (dashed lines).

In contrast to M-Ph-*para*-CF_3_, the dielectric loss spectra of two other sizable systems reveal a strong secondary β-relaxation being distinguishable above and below the glass transition temperature, *T*_*g*_, which is assigned to the internal rotation of the polar segment (Ph-CF_3_). Its properties in terms of relaxation time and dielectric strength in relation to the α-peak are very different. The characteristic relaxation times are shown in Figure 3a–c and were calculated from the fitting parameters obtained by the best fit of the *ε″*(*f*) data to the Cole–Cole function, Havriliak–Negami function, or their superposition.^4^

**Figure 3:**
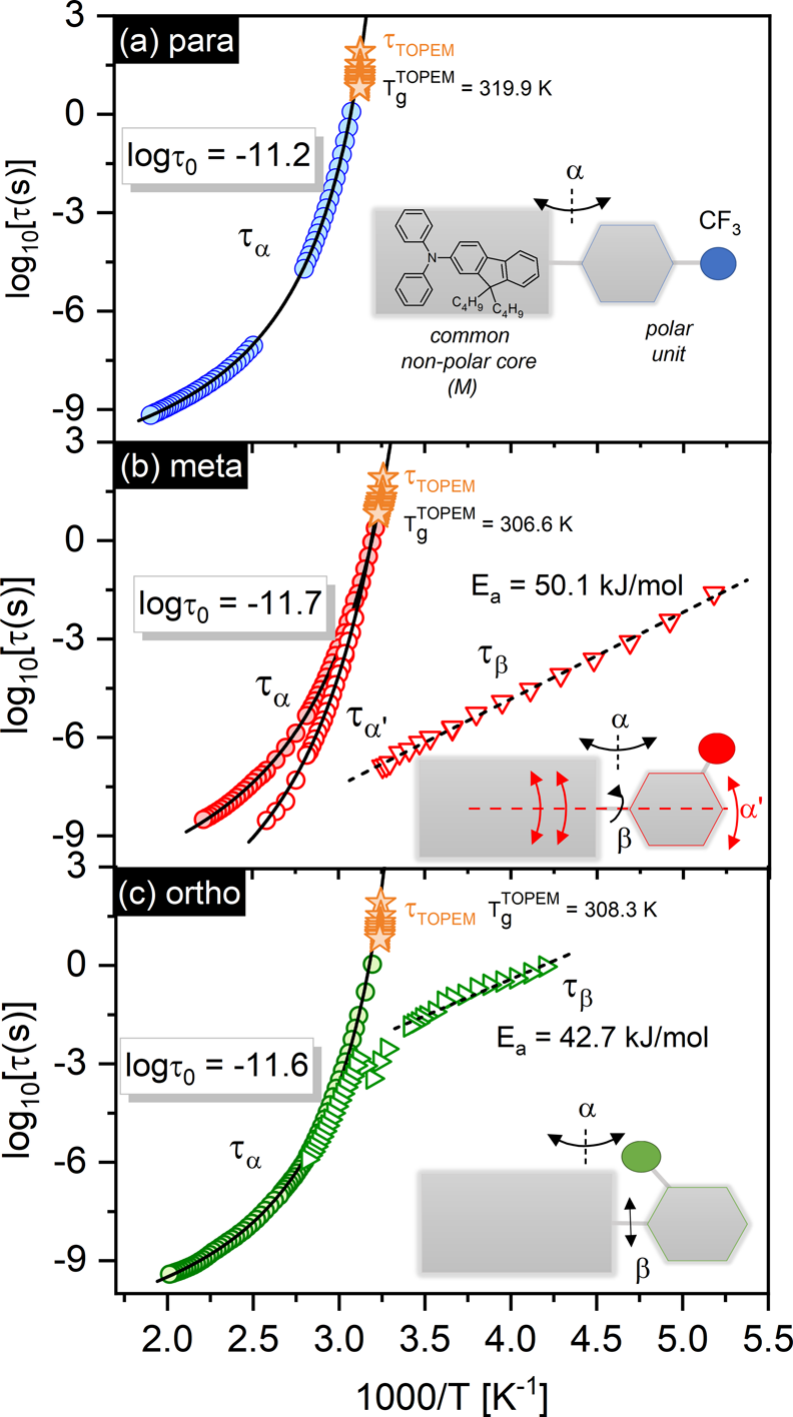
Characteristic relaxation times (circles, τ_α_ or τ_α′_, triangles, τ_β_) plotted versus inverse temperature for *para* (a), *meta* (b), and *ortho* isomers (c) of sizable glass-formers. The solid and dashed lines are fits according to VFT and Arrhenius functions. The values of the VFT pre-exponents τ_0_ are given as characteristic parameters describing their reorientation dynamics. Stars are relaxation times determined from TMDSC measurements (*T*_*g*_^TOPEM^ is indicated). The insets show simplified block schemes of sizable glass-formers demonstrating the observed types of molecular motion.

The reciprocal temperature dependence of the β-relaxation times, τ_β_, for M-Ph-*meta*-CF_3_ and M-Ph-*ortho*-CF_3_ shows the Arrhenius dependence below *T*_*g*_ with the activation energies *E*_*a*_ and prefactors τ_0_ equal to *E*_*a*_ = 50.1 ± 1 kJ/mol and log τ_0_ = −15.4 for M-Ph-*meta*-CF_3_ and *E*_*a*_ = 42.7 ± 2 kJ/mol and log τ_0_ = −9.3 for M-Ph-*ortho*-CF_3_. The substantial overlapping of α*-* and β-peaks in M-Ph-*ortho*-CF_3_ above *T*_*g*_ evidenced that the mechanism of these motions is strongly coupled. As shown in Figure 1b, the loss peak in M-Ph-*ortho*-CF_3_ is the narrowest at high temperatures and widens on cooling, revealing the appearance of a robust β-relaxation peak. The distinct behavior was found for M-Ph-*meta*-CF_3_. On heating, the asymmetry of the main peak gradually increases, showing apart from the β-relaxation an additional contribution to the high-frequency flank of the α-peak. To explore this further, we extended the frequency range up to *f* = 10^9^ Hz and confirmed that at high *T* the main loss peak in M-Ph-*meta*-CF_3_ splits into two relaxation processes. The individual contributions to the dielectric response of the *meta* isomer at *T* = 355 K are unraveled in Figure 2. The observed splitting of the loss peak at high *T* evidences the decreasing degree of cooperativity, which allows for the manifestation of the individual character of molecular motion. On approaching *T*_*g*_, the correlation length ζ increases, leading to the stronger coupling of the dynamics of a particular liquid’s subunits.^10^ The data in Figure 3b portray how the nature of the motions in M-Ph-*meta*-CF_3_ changes on cooling, evolving from a situation where the mobility of each large molecule is independent to a highly intermolecular cooperative behavior. An exceptionally interesting point is a remarkable difference in the character of temperature changes of the relaxation times observed for fast processes, τ_α′_ and τ_β_. At the same time, when the β-process separates from the α-peak as the system cools down, the merging of α- and α′-processes occurs, confirming the principally different origins of both relaxations. Our interpretation of α- and α′-modes in M-Ph-*meta*-CF_3_ takes into account the anisotropic shape of a molecule and is based on two premises: (i) that the movements around the short molecular axis are expected to be slower than those about the long axis and (ii) that the transverse component of the dipole moment is expected to be greater than the longitudinal (this can be deduced from simple trigonometric analysis). Thus, we assigned the slower α-process with higher intensity to the fluctuations of the transverse component of the dipole moment. This component may relax through the whole-molecule reorientations around the short axes that have a larger moment of inertia due to stronger coupling with the mass centers. A faster α′-process was associated with the relaxation of the longitudinal component of the dipole moment by precession about the long axes. Such relaxation needs to be regarded as a combination of two elementary motions involving a sizable but nonpolar molecular core and a much less volumetric but rapidly spinning polar part. Due to the limited rotational freedom of the polar segment in the M-Ph-*ortho*-CF_3_ and its strong coupling with the reorientations of the rest of the sizable molecule, the slowing down of α′- and β-modes compared to M-Ph-*meta*-CF_3_ can be expected. Thus, a simpler relaxation pattern found for the *ortho* isomer can be related to the proximity of the α- and α′-process time scales, which makes both modes experimentally indistinguishable.

Another interesting observation is the lack of fast relaxation processes in the dielectric loss spectra of the M-Ph-*para*-CF_3_. The lack of fingerprints of internal rotations of Ph-CF_3_ in the *para* isomer results from the arrangement of the dipole moment in this system. In M-Ph-*para*-CF_3_, the dipole moment is directed parallel to the long axis of the sizable molecule (the transverse component of the dipole moment vector is negligible), and it does not change during rotation of the Ph-CF_3_ fragment. For the same reason, the frequency dispersion of α-relaxation in M-Ph-*para*-CF_3_ is the narrowest among the investigated sizable systems. As presented in Figure 1a–c, the fits of the α-loss peaks by the Fourier transform of the Kohlrausch–Williams–Watts (KWW) equation,^11,12^ φ(*t*) = exp[−(*t*/τ_α_)^βKWW^ yields β_KWW_ = 0.52 at 313 K for M-Ph-*ortho*-CF_3_, β_KWW_ = 0.60 at 315 K for M-Ph-*meta*-CF_3_, and β_KWW_ = 0.75 at 331 K for M-Ph-*para*-CF_3_. The substantially smaller frequency dispersion of the α-relaxation in M-Ph-*para*-CF_3_ demonstrated by the highest β_KWW_ values is a consequence of the inability of the longitudinally arranged dipole moment in the *para* isomer to capture all aspects of the molecule’s motions. This remarkable observation is in line with our previous results for other representatives of sizable glass-formers where the dipole moment probing the dynamics is affixed to a very small fragment of the large molecule.^7^ Hence, the natural question is how accurately it reproduces the dynamics of the entire system. Our results show that, in the sizable glass-forming molecules, the frequency dispersion of structural relaxation, quantified by β_KWW_ values, is strongly related to the arrangement of the dipole moment, impacting the ability to detect all aspects of molecular motion.

The results presented so far prove that the complex and diverse relaxation pattern of sizable glass-formers revealed by dielectric studies is the product of the following factors: (i) the location of the probe (dipole) and , (ii) its rotational freedom and degree of coupling with nonpolar subunit. To definitely distinguished sizable systems from other classes of glass-forming liquids, we have to return to the analysis of τ_α_(*T*) dependences employing the VFT function in Figure 3a–c, i.e., τ_α_(*T*) = τ_0_ exp[*DT*_0_/(*T* – *T*_0_)],^13−15^ with fitting parameters equal to τ_0_ = 5.7 × 10^–12^ s, *D* = 4.1, and *T*_0_ = 281.3 K for the *para*, τ_0_ = 2.0 × 10^–12^ s, *D* = 5.2, and *T*_0_ = 262.4 K for the *meta*, and τ_0_ = 2.5 × 10^–12^ s, *D* = 4.0, and *T*_0_ = 273.7 K for the *ortho* isomer. One of the most exciting and unique observations coming from our study is that the values of pre-exponential factors in the VFT fit to τ_α_(*T*) are substantially greater than those observed typically for ordinary glass-forming liquids where τ_0_ is phonon-like in a time scale ∼ 10^–14^ s and weakly depends on the nature of the material (see the comparison with propylene carbonate in Figure S1).^16^ The unusually large VFT prefactors (τ_0_ > 10^–12^) established for reorientation dynamics by dielectric measurements are universal for sizable systems tested so far.^7,8^ Therefore, they can be regarded as a characteristic feature distinguishing sizable molecules from other classes of glass-forming liquids. This unique behavior can be rationalized by the effect of inertia. To determine *I* values, we performed DFT calculations (see Supporting Information for more details). For propylene carbonate, *I* = 4.90 × 10^–45^ kg·m^2^, while for M-*para*-CF_3_, *I* = 3.19 × 10^–43^ kg·m^2^. These values yielded τ_0_ = (2π*I*/*k*_B_*T*)^0.5^, which is 8 times longer for a sizable system compared to low-molecular-weight glass-former propylene carbonate (Table S2). In sizable molecules, due to the relevance of inertial effects, we “gain greater resolution” and a unique insight into hitherto unexplored aspects of the reorientation motion of large and anisotropic glass-forming systems probed by the dielectric method.

In summary, our dielectric study of three structural isomers belonging to the latterly constituted class of sizable glass-formers revealed a surprisingly impressive and notable dielectric behavior associated with the different positions of the single dipole moment in a large molecule. Parallel to the spectacular variations in the distribution of structural relaxation times among systems “differently labeled” with the dipole moment, some basic phenomena were discovered related to the onset of inertia, collectivity, and the degree of coupling of an individual subunit’s motion. In general terms, the concept of sizable molecules, being systematized in this Letter, concerns chemical entities being a collection of multiple rigid or semirigid frameworks functionalized with floppy elements (like alkyl solubilizing groups) and small polar units that have properties suitable for many attractive applications. In particular, fluorene-based compounds combining different π-conjugated building blocks are attractive candidates for various light-emitting applications, e.g., in organic light-emitting diodes (OLEDs),^17^ which could fill the gap of today and future demands of flexible electronics. The correlated dynamics of several moving parts within a single molecule allow an exciting extrapolation to the complex artificial machines and biomolecular systems, making sizable systems a platform of materials providing an intriguing starting point for these intricate objects. The results presented in this Letter and the thought-provoking ideas behind them are fascinating due to the possibility of introducing the fundamental issues untouched so far and directing dielectric research into new, hitherto unexplored areas of practical implementation.

## Supporting Information Available

Experimental protocols, summary of the dynamic parameters determined from dielectric measurements, rotational constants, and calculated moments of inertia, and comparison of dielectric responses for sizable molecules and propylene carbonate (PDF)
