# Supplementary material for: Complex Reorientation Dynamics of Sizable Glass-Formers with Polar Rotors Revealed by Dielectric Spectroscopy
Source: J Phys Chem Lett. 2021 Nov 15;12(46):11303–7. doi: 10.1021/acs.jpclett.1c03088 (PMC8630796; doi:10.1021/acs.jpclett.1c03088)
Supplement: Supplementary file 1 — jz1c03088_si_001.pdf [file jz1c03088_si_001.pdf]

Supplemental Material

Complex Reorientation Dynamics of Sizable Glass-Formers with Polar Rotors Revealed by Dielectric Spectroscopy

Marzena Rams-Baron[1,2,*], Beibei Yao[1,2], Shinian Cheng[1,2], Mateusz Dulski[2,3], Marian Paluch[1,2]

[1]*August Chełkowski Institute of Physics, University of Silesia, 75 Pulku Piechoty 1, 41-500 Chorzow, Poland*

[2]*Silesian Center for Education and Interdisciplinary Research, 75 Pulku Piechoty 1a, 41-500 Chorzow, Poland*

[3]*Institute of Materials Engineering, University of Silesia, 75 Pulku Piechoty 1a, 41-500 Chorzow, Poland*

I. Experimental details

1. Broadband Dielectric Spectroscopy (BDS)

Dielectric measurements in the frequency range from 10^{-1} up to 10^6 Hz were performed using a Novocontrol GMBH Alfa impedance analyzer. In the high-frequency measurements (10^6 to 10^9 Hz), we applied Agilent 4291B impedance analyzer connected with the Novocontrol GMBH system. The measurements up to 10^6 Hz were performed in a parallel plate steel capacitor (15 mm diameter) with a fixed distance between electrodes (0.1 mm) provided by fused silica fibers. During the measurements in a high-frequency regime, the sample was placed between two gold-plated electrodes (diameter, 5 mm; gap, 0.06 mm). The samples were prepared by the melt quench cooling method. The capacitor with the investigated sample was heated up to the melting temperature (T_m = 443 K for M-Ph-*para*-CF_3, and T_m = 385 K for M-Ph-*meta*-CF_3, and T_m =399 K for M-Ph-*ortho*-CF_3) and then was quenched by a fast transfer to a precooled copper plate. BDS measurements were performed in a wide temperature range corresponding to the glassy, supercooled liquid and normal liquid states. Quatro Cryosystem precisely controlled the temperature with high accuracy of 0.1 K.

* Author to whom correspondence should be addressed: marzena.rams-baron@us.edu.pl

2. Temperature-Modulated Differential Scanning Calorimetry (TMDSC)

The TMDSC experiments were carried out using a Mettler Toledo DSC1STAR System with a liquid nitrogen cooling accessory and an HSS8 ceramic sensor, a heat flux sensor with 120 thermocouples. The enthalpy and temperature calibrations of the system were performed using indium and zinc standards. The samples were measured in the aluminum crucibles with a volume of 40 μL. The calorimetric measurements were performed on heating with a rate of 0.5 K min^{-1}, and the temperature amplitude of the pulses was 1 K. The experimental temperature range was from 233.15 K to 473.15 K. During the experiments, the nitrogen flow was keeping at a constant rate of 60 mL min^{-1}. The standard sapphire curve was applied to calibrate the obtained TMDSC curves. Using a stochastic TMDSC technique, the dynamic behavior of the glass transition of measured compounds was analyzed in the frequency range from 2 to 25 mHz.

3. DFT calculations of rotational constants

To determine the moment of inertia for sizable molecules we calculated rotational constants B for axes labeled as x, y, z (conventionally chosen to be in the order of increasing values of the principal moments of inertia). The initial geometries of sizable molecules were optimized using the molecular modelling method implemented in the Gaussian09 software package.[1] The calculations were performed for an isolated molecule using density functional theory (DFT) in the gas phase[2],[3],[4] at B3LYP functional[5],[6] and split-valence 6-31+G(d, p) basis sets with diffuse d as well as p functions. The harmonic vibrations for all molecules were calculated, wherein positive values prove a true energy minimum of the system.

II. Results:

Table S1. The summary of dynamic parameters which were determined using the BDS method.

	BDS					
	T_g [K][a]	fragility m[b]	VFT parameters			β_{KWW}
			τ_0 [s]	D	T_0 [K]	
M-Ph-*para*-CF_3	319.0	113	$5.7 \cdot 10^{-12}$	4.1	281.3	0.75
M-Ph-*meta*-CF_3	305.7	97	$2.0 \cdot 10^{-12}$	5.2	262.4	0.60
M-Ph-*ortho*-CF_3	308.8	120	$2.5 \cdot 10^{-12}$	4.0	273.7	0.52

[a] Determined from the VFT fits as $\tau_\alpha(T_g) = 100$ s

[b] Determined from the VFT fits as $m = d\log(\tau_\alpha)/d(T_g/T)|_{T=T_g}$

Table S2. Rotational constants B [GHz] obtained from DFT calculations for sizable systems and canonical low-molecular-weight glass-former propylene carbonate (PC), and calculated moments of inertia I and theoretical τ_0 values determined from Bauer expression.

	**Rotational constant** B [GHz]			**Moment of inertia** I [kg·m^2]^a			τ_0 **[s]**^{b} calculated for z-axis
	x	y	z	x	y	z	
PC	5.966	2.214	1.716	$1.41\cdot10^{-45}$	$3.80\cdot10^{-45}$	$4.90\cdot10^{-45}$	$2.9\cdot10^{-12}$
M-Ph-*ortho*-CF_3	0.130	0.038	0.034	$6.47\cdot10^{-44}$	$2.24\cdot10^{-43}$	$2.46\cdot10^{-43}$	$2.0\cdot10^{-11}$
M-Ph-*meta*-CF_3	0.130	0.029	0.029	$6.46\cdot10^{-44}$	$2.89\cdot10^{-43}$	$2.95\cdot10^{-43}$	$2.2\cdot10^{-11}$
M-Ph-*para*-CF_3	0.144	0.028	0.026	$5.85\cdot10^{-44}$	$3.01\cdot10^{-43}$	$3.19\cdot10^{-43}$	$2.3\cdot10^{-11}$

^{a} calculated as $I = h/(8\pi^2 cB)$ where h is Planck's constant and c is the speed of light

^{b} calculated as $\tau_0 = (2\pi I/k_B T)^{0.5}$ for T = 273 K.

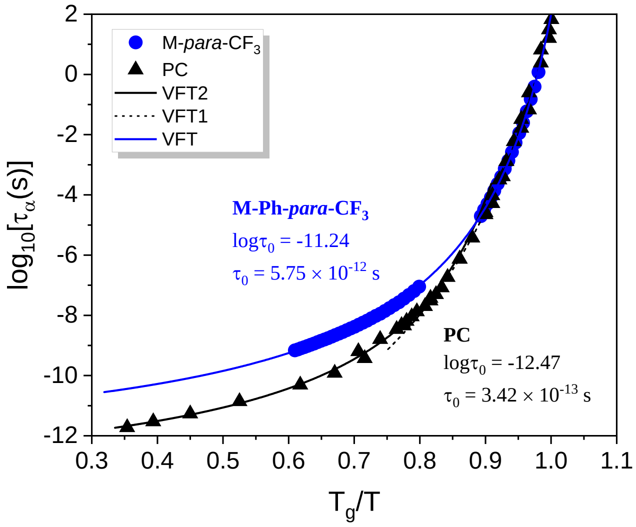

Fig. S1. The differences in the character of $\log\tau_\alpha(T_g/T)$ and in the high-temperature limits of VFT fits between the sizable system M-Ph-*para*-CF_3 and low-molecular-weight glass-former propylene carbonate (PC). For PC experimental data were taken from ref. [7].

References:

(1) Frisch, M. J.; Trucks, G. W.; Schlegel, H. B.; Scuseria, G. E.; Robb, M. A.; Cheeseman, Gaussian 09, Revision A.02. **2016**.

(2) Hehre, W. J.; Radom, L.; Schleyer, P. R.; Pople, J. *Ab Initio Molecular Orbital Theory*; John Wiley: New York, 1989.

(3) Parr, R. G.; Weitao, Y. *Density Functional Theory Of Atoms and Molecules; Oxford*

Univ. Press: New York, 1994.

(4) Dobson, J. F.; Vignale, G.; Das, P. *Electronic Density Functional Theory: Recent Progress and New Directions*; Springer: New York, 1998.

(5) Becke, A. D. Density-Functional Exchang-Energy Approximation with Correct Asymptotic Behavior. *Phys. Rev. A* **1988**, *38* (6), 3098–3100.

(6) Becke, A. D. Density-Functional Thermochemistry. III. The Role of Exact Exchange. *J. Chem. Phys.* **1993**, *98* (7), 5648–5652.

(7) Schmidtke, B.; Petzold, N.; Kahlau, R.; Rössler, E. A. Reorientational Dynamics in Molecular Liquids as Revealed by Dynamic Light Scattering: From Boiling Point to Glass Transition Temperature. *J. Chem. Phys.* **2013**, *139* (8), 084504.
